# Evolution by duplication: paleopolyploidy events in plants reconstructed by deciphering the evolutionary history of VOZ transcription factors

**DOI:** 10.1186/s12870-018-1437-8

**Published:** 2018-10-26

**Authors:** Bei Gao, Moxian Chen, Xiaoshuang Li, Yuqing Liang, Fuyuan Zhu, Tieyuan Liu, Daoyuan Zhang, Andrew J. Wood, Melvin J. Oliver, Jianhua Zhang

**Affiliations:** 1 School of Life Sciences and the State Key Laboratory of Agrobiotechnology, The Chinese University of Hong Kong, Hong Kong, China; 2 Shenzhen Research Institute, The Chinese University of Hong Kong, Shenzhen, China; 3 Key Laboratory of Biogeography and Bioresources, Xinjiang Institute of Ecology and Geography, Chinese Academy of Sciences, Urumqi, 830011 China; 4 College of Biology and the Environment, Nanjing Forestry University, Nanjing, Jiangsu Province, 210037 China; 5 Department of Plant Biology, Southern Illinois University-Carbondale, Carbondale, IL 62901-6509 USA; 6 USDA-ARS, Plant Genetic Research Unit, University of Missouri, Columbia, MO 65211 USA; 7 Department of Biology, Faculty of Science, Hong Kong Baptist University, Hong Kong, China

**Keywords:** Polyploidy, Whole genome duplication, Transcription, Plant evolution, Gamma

## Abstract

**Background:**

Facilitated by the rapid progress of sequencing technology, comparative genomic studies in plants have unveiled recurrent whole genome duplication (i.e. polyploidization) events throughout plant evolution. The evolutionary past of plant genes should be analyzed in a background of recurrent polyploidy events in distinctive plant lineages. The **V**ascular Plant **O**ne **Z**inc-finger (VOZ) gene family encode transcription factors associated with a number of important traits including control of flowering time and photoperiodic pathways, but the evolutionary trajectory of this gene family remains uncharacterized.

**Results:**

In this study, we deciphered the evolutionary history of the VOZ gene family by analyses of 107 *VOZ* genes in 46 plant genomes using integrated methods: phylogenic reconstruction, *Ks*-based age estimation and genomic synteny comparisons. By scrutinizing the *VOZ* gene family phylogeny the core eudicot γ event was well circumscribed, and relics of the precommelinid τ duplication event were detected by incorporating genes from oil palm and banana. The more recent *T* and ρ polyploidy events, closely coincident with the species diversification in Solanaceae and Poaceae, respectively, were also identified. Other important polyploidy events captured included the “salicoid” event in poplar and willow, the “early legume” and “soybean specific” events in soybean, as well as the recent polyploidy event in *Physcomitrella patens*. Although a small transcription factor gene family, the evolutionary history of *VOZ* genes provided an outstanding record of polyploidy events in plants. The evolutionary past of VOZ gene family demonstrated a close correlation with critical plant polyploidy events which generated species diversification and provided answer to Darwin’s “abominable mystery”.

**Conclusions:**

We deciphered the evolutionary history of VOZ transcription factor family in plants and ancestral polyploidy events in plants were recapitulated simultaneously. This analysis allowed for the generation of an idealized plant gene tree demonstrating distinctive retention and fractionation patterns following polyploidy events.

**Electronic supplementary material:**

The online version of this article (10.1186/s12870-018-1437-8) contains supplementary material, which is available to authorized users.

## Background

The evolutionary history of land plants is characterized by recurrent polyploidy (whole genome duplication, WGD) events, which provided novel genetic materials and contributed heavily to the species diversification process, thus WGD events are regarded as important driving forces in evolution [1–4]. Facilitated by the high-throughput sequencing technology, the completion of more and more plant genome sequences and advances in comparative genomic methods led to an acceleration in the identification of recurrent polyploidy events in different plant lineages [5–8].

Two ancestral polyploidy events were identified using phylogenomic approaches, one of which affected all seed plants (termed ξ, ~ 319 Mya) and another one that can be seen in all angiosperms (termed ε, ~ 192 Mya) [9, 10]. In the eudicots, representing over 75% of extant angiosperms, the γ whole genome triplication event occurred around 117 Mya and is associated with the early diversification of the core eudicots. The γ whole genome triplication event occurred after the divergence of Ranunculales [11], then placed precisely before the separation of Gunnerales but after the divergence of Buxales and Trochodendrales by more detailed analyses [12]. Based on age distributions and chromosome structural analyses with fully sequenced genomes, a series of recurrent polyploidy events have been identified [5, 8]. For example, in the *Arabidopsis thaliana* genome, three recurrent polyploidizations constituting the α-β-γ WGD series were detected [6] and in *Populus* and *Salix* the “salicoid” duplication event (alternatively termed *p*) was discovered as a shared WGD prior to speciation [13–15], thus constituting the “salicoid”-γ WGD series for Salicaceae. In the agriculturally and economically important soybean (*Glycine max*) genome another two paleopolyploidy events following the γ event were identified and formed the “soybean specific”-“early legume”-γ WGD series [16, 17]. In the asterid lineage, both potato and tomato genomes contained evidence for a common *Solanum* whole genome triplication event (termed *T*) and formed the *T*-γ polyploidization series in *Solanum* [18, 19]. A unique polyploidy event (termed λ) occurred in the genome of the basal eudicot sacred lotus (*Nelumbo nucifera*). The lotus-specific λ WGD event occurred about 65 Mya and its genome lacks the footprint of the γ hexaploidy event [20].

In monocots, echoing the α-β-γ WGD series in *Arabidopsis,* the *Oryza* and other grass genomes have also experienced three recurrent polyploidy events, constituting the ρ-σ-τ WGD series [21–23], where the τ event was estimated to have occurred before the separation of Arecaceae and Poaceae, the recurrent ρ and σ WGD events took place after τ. Two polyploidy events were discovered in the genome of oil palm (*Elaeis guneensis,* Arecaceae) which correspond to the p-τ WGD events [21, 22, 24–26].

As a sister lineage to angiosperms, the first conifer genome in Norway spruce (*Picea abies*), reported the presence of a WGD with a *Ks* peak at ~ 1.1, but somehow overlooked another peak consistent with a WGD near *Ks* ~ 0.25 [27]. A more recent systemic study in conifers identified two WGD events in the ancestry of the major conifer clades (Pinaceae and cupressophyte conifers) and in Welwitschia (Gnetales) [28]. For bryophytes, the genome of the model moss *Physcomitrella patens* also indicated a large-scale genome duplication with conspicuous *Ks* peak around 0.5–0.9 [29], whereas more ancient WGD events in mosses and bryophytes remain elusive.

Polyploidization provided crucial evolutionary materials and functional novelty for plant evolution and was frequently followed by diploidization. Diploidization involves both extensive silencing and elimination of duplicated genes (fractionation) [30–32] besides gene retention. Retention of duplicated genes was demonstrated to be functionally biased as dosage balance-sensitive genes [33], such as transcription factors, are significantly over-retained following WGDs [34]. For example, in the *Arabidopsis* genome, gene retention following the most recent α (3R) polyploidy event is much lower and less functionally biased compared to the γ (1R) and β (2R) events and all three polyploidy events together contributed directly to more than 90% of the increase in transcription factor genes [2, 35].

Of all transcription factors, the evolutionary history of the MADS-box transcription factor family has been the most widely studied [36–44]. This is in large part due to their roles in flower development and as dominant components of the “ABCDE model” [1, 45–47]. Several subfamilies of MADS-box genes have duplicated or triplicated during their evolutionary past. Additionally, along with the evolution of MADS-box gene family per se [12, 41], the protein-protein interaction (PPI) network among MADS-box genes in basal eudicots [48] have also been investigated. The fine-tuning of flowering time is clearly critical for angiosperm development and reproduction as well as the fitness and fate of a species in history, it is for this reason that the evolution of TF gene families in these developmental pathways is of particular interest.

In the Flowering Interactive Database (FLOR-ID, http://www.phytosystems.ulg.ac.be/florid/), a list of 306 flowering time genes in *Arabidopsis* were recorded. These flowering time genes can be assigned to four interlocking flowering pathways: “photoperiodic”, “vernalization”, “autonomous” and “gibberellin” pathways [49, 50]. Within the “photoperiodic pathway” two VASCULAR PLANT ONE-ZINC FINGER (*VOZ)* genes were first identified and characterized in *Arabidopsis*, and homologs in rice and the moss *P. patens* were also identified [51]. The two *VOZ* genes in *Arabidopsis* regulate flowering time by interacting with phytochrome B and FLC. The two genes act in a redundant fashion as only double-mutants exhibit late flowering phenotypes under long-day conditions [52–54]. *VOZ* genes are also involved in abiotic and biotic stress responses [55, 56].

As a flowering-time regulatory transcription factor family that is apparently well conserved in land plants [57], the origin and evolutionary history of *VOZ* genes in plants is of biological significance.

In this study, we revealed and reconstructed multiple nested lineage- and species-specific polyploidy events in plants (e.g. the γ event in eudicots, τ in commelinids, *T* in Solanaceae and ρ in grasses) by deciphering the evolutionary history of VOZ transcription factor family in 46 plant genomes. This was achieved by utilizing an integrated approach that included phylogenic reconstructions, molecular dating and genomic collinearity analyses. *In toto*, the evolutionary history of VOZ transcription factor family presented here represents a robust case in which unambiguous paralogous and orthologous relationships were well resolved and provided a concise and logical framework for the identification and placement of the well-known polyploidy events that shaped multiple plant lineages.

## Results

### Phylogenic analyses, classification and nomenclature

**Fig. 1:**
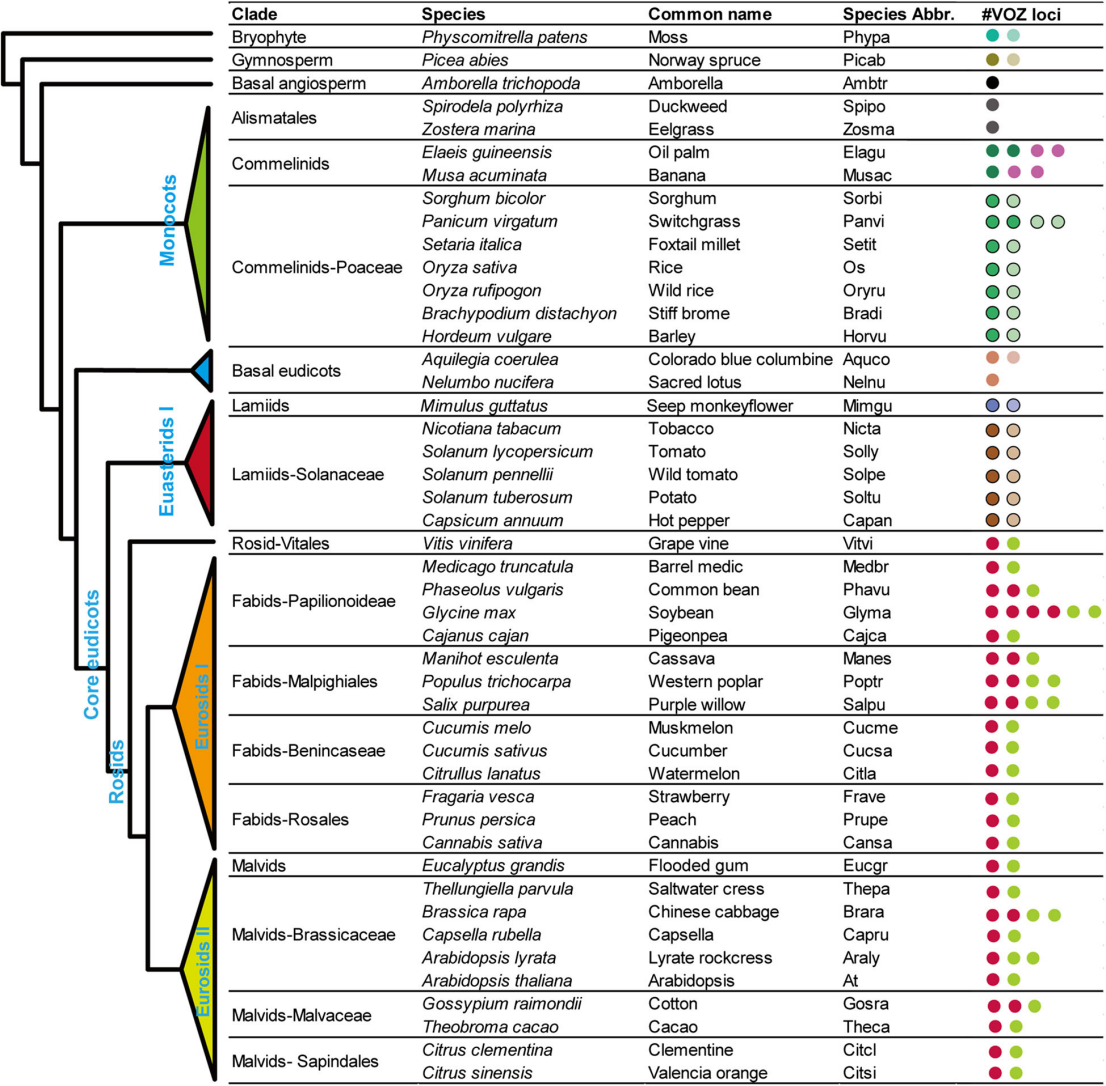
Inventory of analyzed plant *VOZ* transcription factor genes in major plant linages. The number of dots represents the number of *VOZ* genes in the genome. Dots with the same color represent members that belong to the same subfamily

To elucidate its evolutionary history, we collected a total of 107 VOZ transcription factors from 46 plants for which genome sequences were available (Additional file 1: Table S1). Representatives from each of the dominant plant lineages were incorporated in the analysis: including one bryophyte (*Physcomitrella patens*), one gymnosperm (*Picea abies*), one basal angiosperm (*Amborella trichopoda*), eleven monocot species (seven of which were grasses), and 32 eudicots (two basal eudicots, six asterids, thirteen fabids, ten malvids and *Vitis vinifera*). The VOZ transcription factor was demonstrated to be a conserved small gene family with one to six members (Fig. 1). As recorded in PlantTFDB [57], VOZ transcription factors are restricted to the land plants and originally emerged in the genomes of bryophytes but are absent in the liverwort *Marchantia polymorpha* (Marchantiophyta) and the lycophyte *Selaginella moellendorffii* (Lycopodiophyta), which was validated by whole genome homolog sequence searches.

**Fig. 2:**
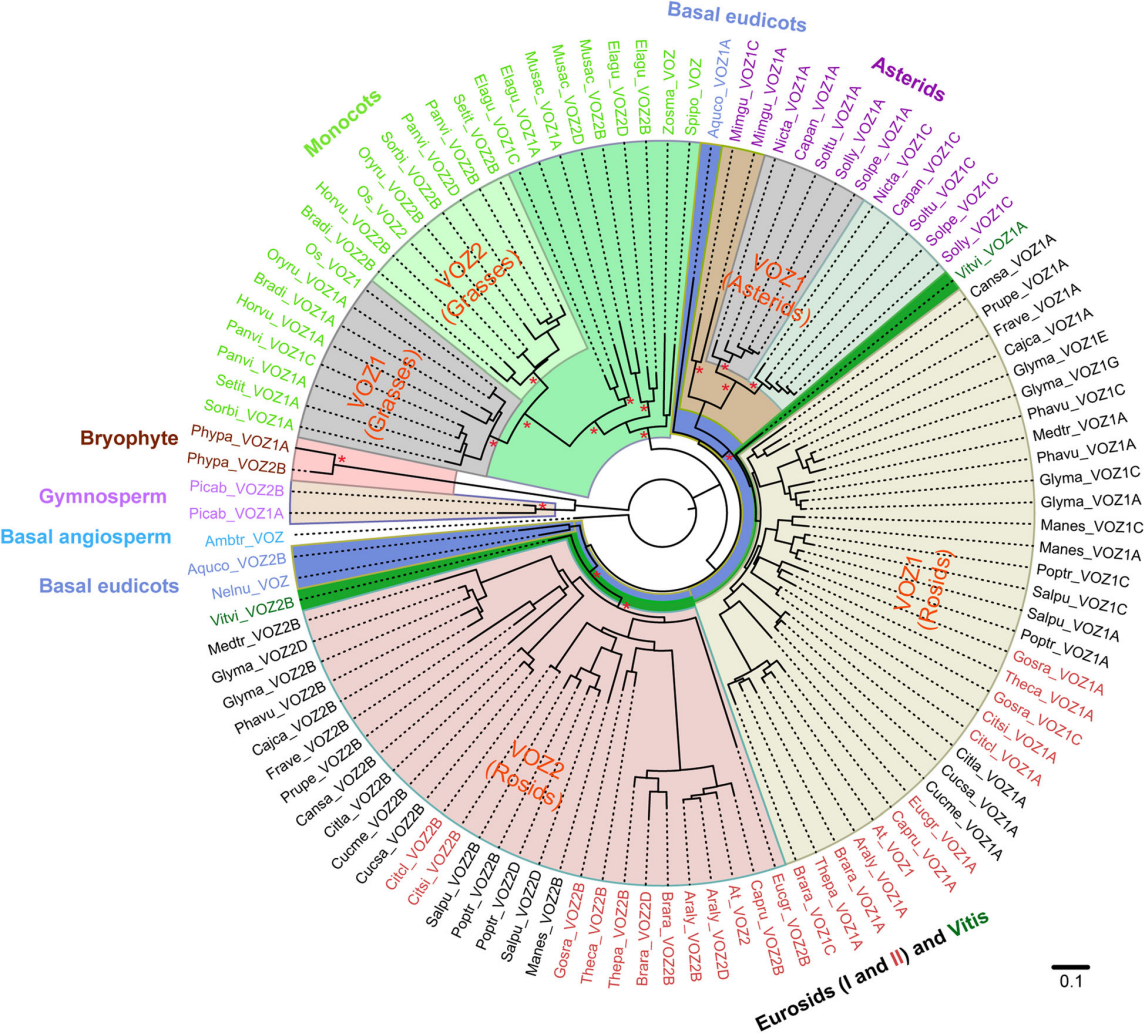
The phylogenic tree of plant *VOZ* transcription factor genes. The best representative Maximum-likelihood (ML) topology generated with the GTR + G + I model using RAxML for the 107 VOZ transcription factor coding sequences. Branch lengths indicate the number of nucleotide substitutions per site and are drawn to scale. All species abbreviations are listed in Fig. 1. A phylogenic tree reconstructed utilizing the Bayesian Inference (BI) method in MrBayes exhibits a similar topological structure (Additional file 2: Figure S1). Asterisks indicate the focal nodes were well supported in both RAxML (bootstrap values of 90) and MrBayes (posterior probability support of 95%). Individual clades are colored for ease of visualization

For phylogenic analyses, the protein guided coding sequence alignments were automatically trimmed. Unrooted gene trees were constructed from the multiple sequence alignments, with both the Maximum Likelihood (ML) method using RAxML (Fig. 2) and the Bayesian Inference (BI) method using MrBayes (Additional file 2: Figure S1). Gene trees constructed with both methods demonstrated similar topological structures and indicated a highly consistent pattern with various plant lineages. The four *VOZ* genes from the moss and the gymnosperm clustered outside of the angiosperm clade and the unique gene (*Ambtr_VOZ*) from *Amborella trichopoda* was placed sister to all the other angiosperm *VOZ* genes. *Ambtr_VOZ* was subsequently utilized as an ideal single-copy outgroup sequence for all monocot and eudicot lineages.

Within angiosperms, *VOZ* genes could be largely divided into three groups representing monocots, asterids and rosids clades, within which the *VOZ* genes from basal eudicotyledons (*Nelumbo nucifera* and *Aquilegia coerulea*) delineated the boundary of all eudicots, and the two *VOZ* genes of *Vitis vinifera* located sister to all rosid genes. Genes from asterids (mostly represented by the Solanaceae) were clustered outside the VOZ1-Rosids clade, but inside the large clade for eudicots (boundaries indicated by *Aquilegia* and *Nelumbo*). For monocots, dominated by grasses, the grass *VOZ* genes were clustered together because of their close phylogenic relationships, constituting the VOZ-Grasses clade as depicted in Fig. 2. *VOZ* genes from the two commelinids, banana (*Musa acuminata*) and oil palm (*Elaeis guineensis,* Arecaceae), clustered outside the VOZ-Grasses clade and the genes from the two Alismatales (*Spirodela polyrhiza* and *Zostera marina*), both of which are aquatic monocots and possess single-copy *VOZ* genes that constituted a clade sister to the genes from commelinids.

To date, no concise nomenclature reflecting phylogenetic relationships has been developed for the VOZ gene family. We propose a simplified nomenclature procedure for VOZ transcription factors that complies with the lineage- and species-specific genomic duplication events leading to the occurrence of orthologs and paralogs, as described below. This classification is based on phylogenic placement within the gene tree combined with extant classification in previous experimental reports of *VOZ* genes in *Arabidopsis thaliana* [51] and *Oryza sativa* [58], which remain unaltered as *At_VOZ1* (AT1G28520), *At_VOZ2* (AT2G42400), and *Os_VOZ1* (Os01g0753000) and *Os_VOZ2* (Os05g0515700). Generally, in most plant species analyzed, VOZ transcription factors could be classified into two major subfamilies, denoted as VOZ1 and VOZ2 on the phylogenic tree in accordance with the reported members in rice and *Arabidopsis*.

In the phylogenic tree, *VOZ* genes in rosids were split into two clades (i.e. VOZ1-Rosids and VOZ2-Rosids) (Fig. 2). Considering the lineage- or species-specific duplications, genes in the VOZ1-Rosids clade were classified as *VOZ1A, VOZ1C, VOZ1E,* genes present in the VOZ2-Rosids clade were classified as *VOZ2B, VOZ2D, VOZ2F,* … with each gene name prefixed with its five-letter species abbreviation. In many cases, a species contains two VOZ genes. For example, in the grape genome two genes occur in the VOZ1-Rosids and VOZ2-Rosids clades, and the genes were named as *Vitvi_VOZ1A* (VIT_10s0003g00500) and *Vitvi_VOZ2B* (VIT_12s0028g02670), respectively. In the poplar genome, four *VOZ* genes were identified with two members occurring in the VOZ1-Rosids clade and the other two in the VOZ2-Rosids clade, and these genes were classified as *Poptr_VOZ1A* (Potri.004G050900), *Poptr_VOZ1C* (Potri.011G060000), *Poptr_VOZ2B* (Potri.013G123100) and *Poptr_VOZ2D* (Potri.019G092800). *Poptr_VOZ1s* and *Poptr_VOZ2s* reflect the ancestral core eudicot-wide duplication, and paralogous pairs of *Poptr_VOZ1A* vs. *Poptr_VOZ1C*, and *Poptr_VOZ2B* vs. *Poptr_VOZ2D* probably represented products for more recent lineage-specific duplications. For genes in asterids (dominantly represented by Solanaceae species), the paleoparalogs in the “VOZ2-Asterids” clade were not observed as a result of subsequent widespread gene losses [19]. And all the genes in asterids analyzed here were included in the VOZ1-Eudicots clade, so genes in VOZ1-Asterids were basically classified as *VOZ1A* and *VOZ1C*, in congruent with more recent lineage-specific duplications.

Similarly, in the monocot clades, *VOZ* genes from grasses were readily separated into two subfamilies (denoted as VOZ1-Grasses and VOZ2-Grasses) using *Os_VOZ1* and *Os_VOZ2* as anchors (Fig. 2). However, this cannot facilitate the classification of *VOZ* genes in other monocot members because they reside outside the Poaceae clade in the gene tree. Scrutinizing the gene tree topologies in the clade of monocots, signals for a precommelinid duplication followed by a species-specific duplication event were apparent. Thus, the *VOZ* genes from banana and oil palm were named following the rules mentioned above to reflect ancestral gene duplications as depicted in Fig. 2. The genes from banana and oil palm segregate into the cluster sister to the VOZ-Grasses clade and were classified as a VOZ1 subfamily because they demonstrated collinearity with the genomic regions that flank *Os_VOZ1* gene locus. In this scenario, the VOZ-Grasses (including VOZ1-Grasses and VOZ2-Grasses) clade were nested in the VOZ1-commelinids clade. For species that contain a single-copy VOZ transcription factor gene within the genome (i.e. *Amborella trichopoda, Nelumbo nucifera* and two Alismatales (*Spirodela polyrhiza* and *Zostera marina*)), the genes were concisely classified like “*Ambtr_VOZ*” without suffixes. In this way, the membership to the two major subfamilies of VOZ transcription factor becomes apparent in most plants.

### The VOZ gene loci are located in conserved genomic syntenic regions

**Fig. 3:**
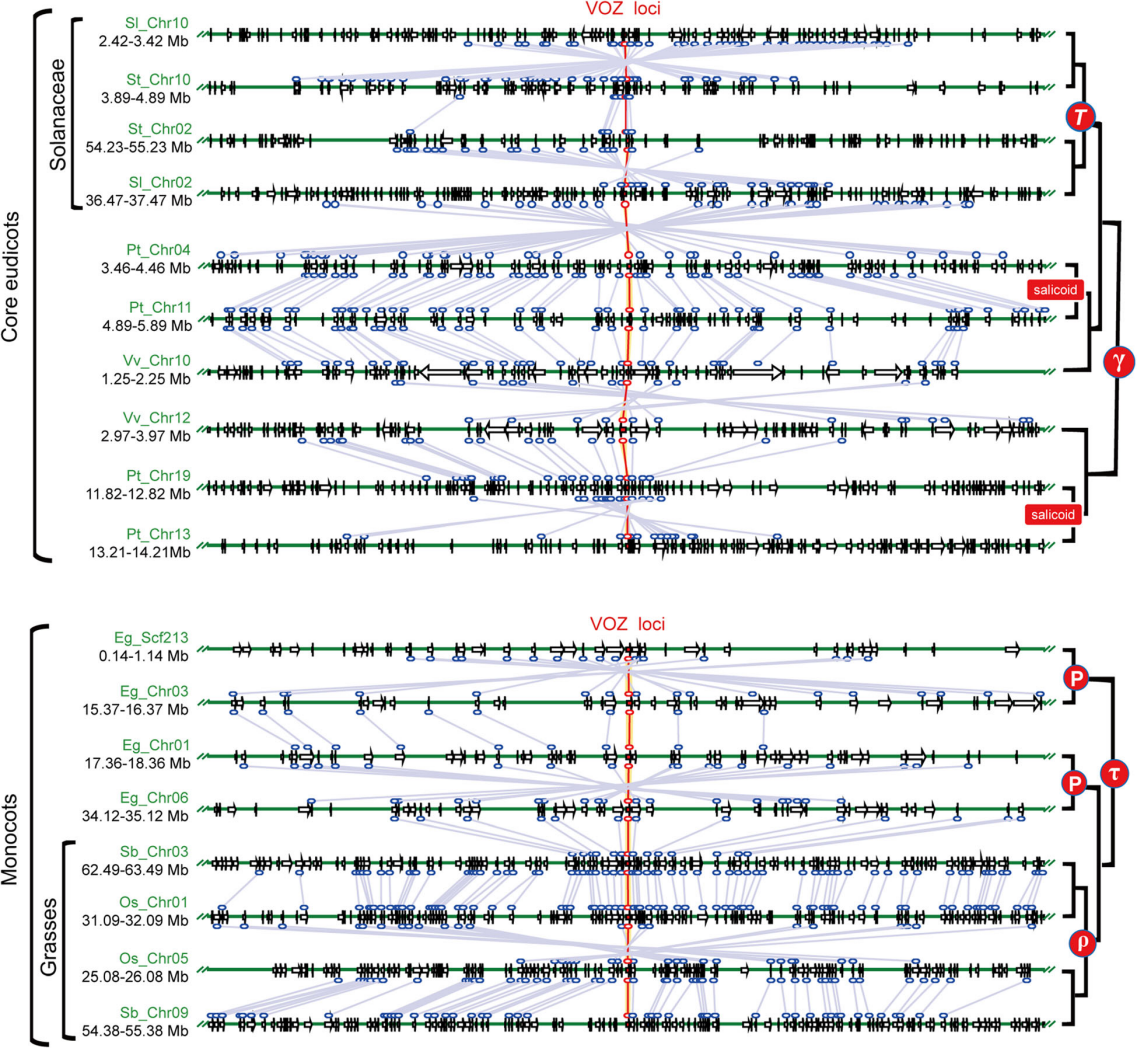
Multiple alignment of the VOZ-containing genomic regions. Analyzed species included tomato (*Solanum lycopersicum*, Sl), potato (*Solanum tuberosum*, St), poplar (*Populus trichocarpa*, Pt), grape (*Vitis vinifera*, Vv), oil palm (*Elaeis guineensis,* Eg), sorghum (*Sorghum bicolor*, Sb) and rice (*Oryza sativa*, Os), related chromosome or scaffold number and coordinates were also indicated. The *VOZ* gene loci were denoted as red arrows and linked by red lines. Arrows in the 1-Mb genomic region represented individual genes and homologs were connected by grey lines. The lineage- or species-specific polyploidy events in monocots and eudicots are indicated in the right panel

To investigate whether the evolution of *VOZ* genes was tightly linked to historical polyploidy events, intra- and inter-species genome alignments centered by the *VOZ* gene loci were performed among three monocots (oil palm, sorghum and rice) and four eudicots (grapevine, poplar, tomato and potato) (Fig. 3). In accordance with the reconstructed phylogenic gene tree, these seven genomes encompass clear evidence for the γ and τ triplication events that occurred in eudicots and monocots respectively, as well as the more recent *T* triplication in asterids, the ρ event in grasses and the “salicoid” event for Salicaceae (right panel in Fig. 3). In the genome of poplar (Pt), the two pairs of chromosomal collinearity following the more recent “salicoid” event were well retained (Pt-Chr 04 and 11 in Fig. 3) presumably because of a much slower evolutionary rate. As representative sister group of all rosids [59], *Vitis* (Vv-Chr10 and 12 in Fig. 3) is the ideal material to trace the ancestral γ event because no subsequent ploidy changes occurred in its genome. In Solanaceae and Poaceae, the genomic synteny blocks flanking the *VOZ* gene loci were well conserved and they were proved as the products of the more recent K-Pg boundary (ca. 65 Mya) polyploidy events [8].

As a complement of the analysis of the conserved genomic synteny in the *VOZ* gene flanking regions, we also examined the gene structure in representative species (Additional file 3: Figure S2). The *VOZ* gene structures were highly conserved with four coding regions interspaced by three introns with intron phases of 0, 0 and 1 respectively. Exceptions were only observed in *Os_VOZ1*, where the first coding region was lost and in *Physcomitrella patens*, where an extra coding region was attached to the 5′ end of the gene. Nevertheless, in all cases the conserved intron phase patterns were retained.

**Fig. 4:**
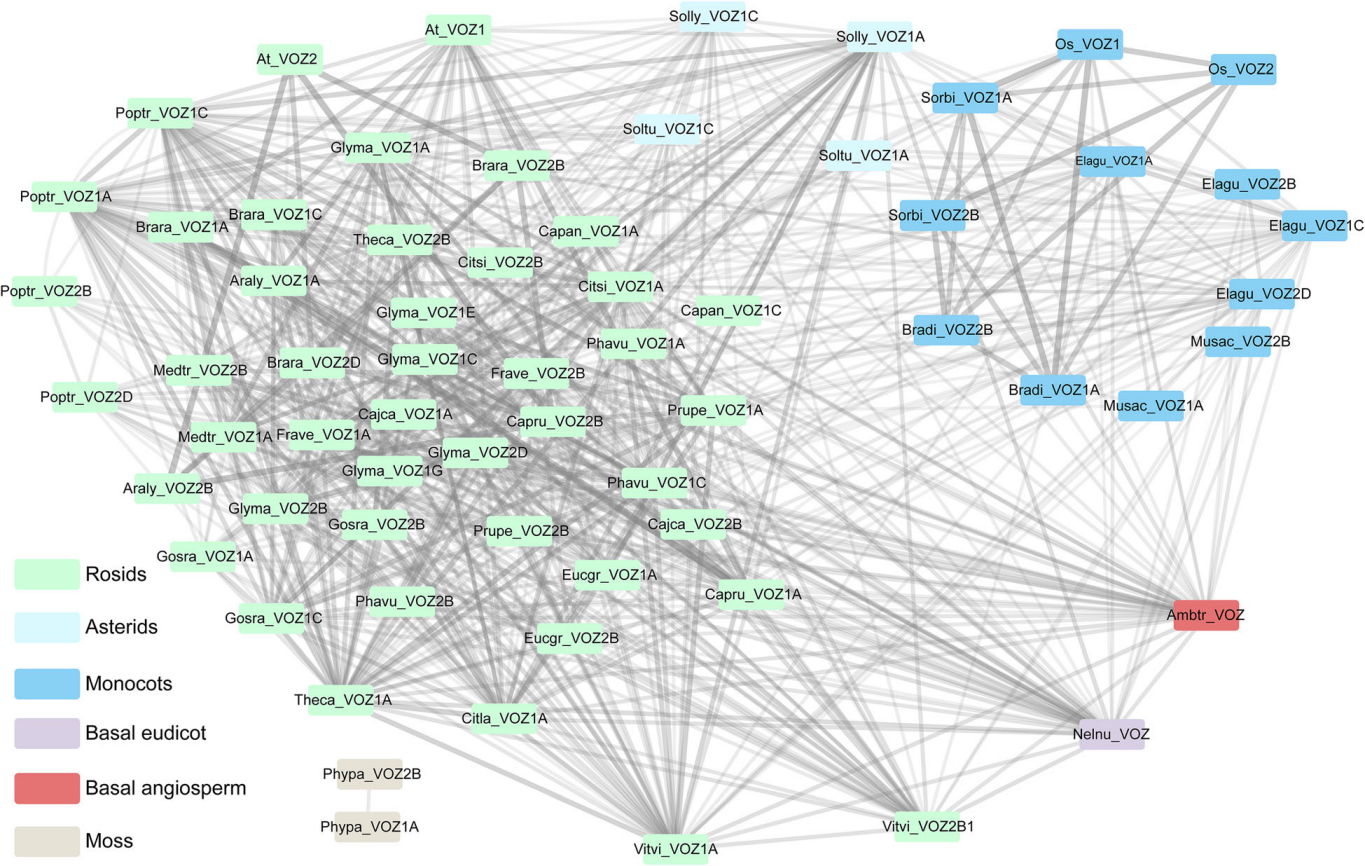
Construction of the syntenic network for plant genomes using the *VOZ* gene loci as anchors. Species abbreviations used in this network are listed in Fig. 1. Pairs of *VOZ* gene loci were connected by lines if located in corresponding syntenic genomic regions. Line weights are in proportion to the syntenic block score (log transformed) calculated by MCScan, where thicker lines largely indicate larger syntenic blocks where the *VOZ* gene loci reside

To illustrate all intra- and inter-genomic synteny relationships among the plant species, a more comprehensive genomic collinearity network associated with the *VOZ* loci was constructed and visualized, with network nodes representing the VOZ-associated genomic regions and edges (lines connecting nodes) indicating the genomic syntenic relationships. Pervasive conserved genomic syntenies could be observed throughout a wide range of species among the angiosperms and in the selected moss. The correlated gene arrangements among taxa provide a valuable framework for inference of shared ancestry of genes. In our analysis, intensive conserved genomic regions within the *VOZ*-containing syntenic blocks were observed, a total of 45 syntenic relationships with other angiosperms were detected for the *Ambtr_VOZ* adjacent genomic region (Fig. 4). The *VOZ* syntenic block in *Amborella* (probably nearest to the ancestral state) shared the most collinearity with other plant genomes than observed in any other species. From this comprehensive syntenic network analysis, it demonstrates that the *VOZ* genes in monocots and eudicots shared a common ancestor and that it is also highly conserved in the genome of *Amborella*, a representative species sister to the rest of the angiosperms.

### *Ks*-based molecular dating of the paleo-polyploidy events using duplicated syntenic paralogs

**Fig. 5:**
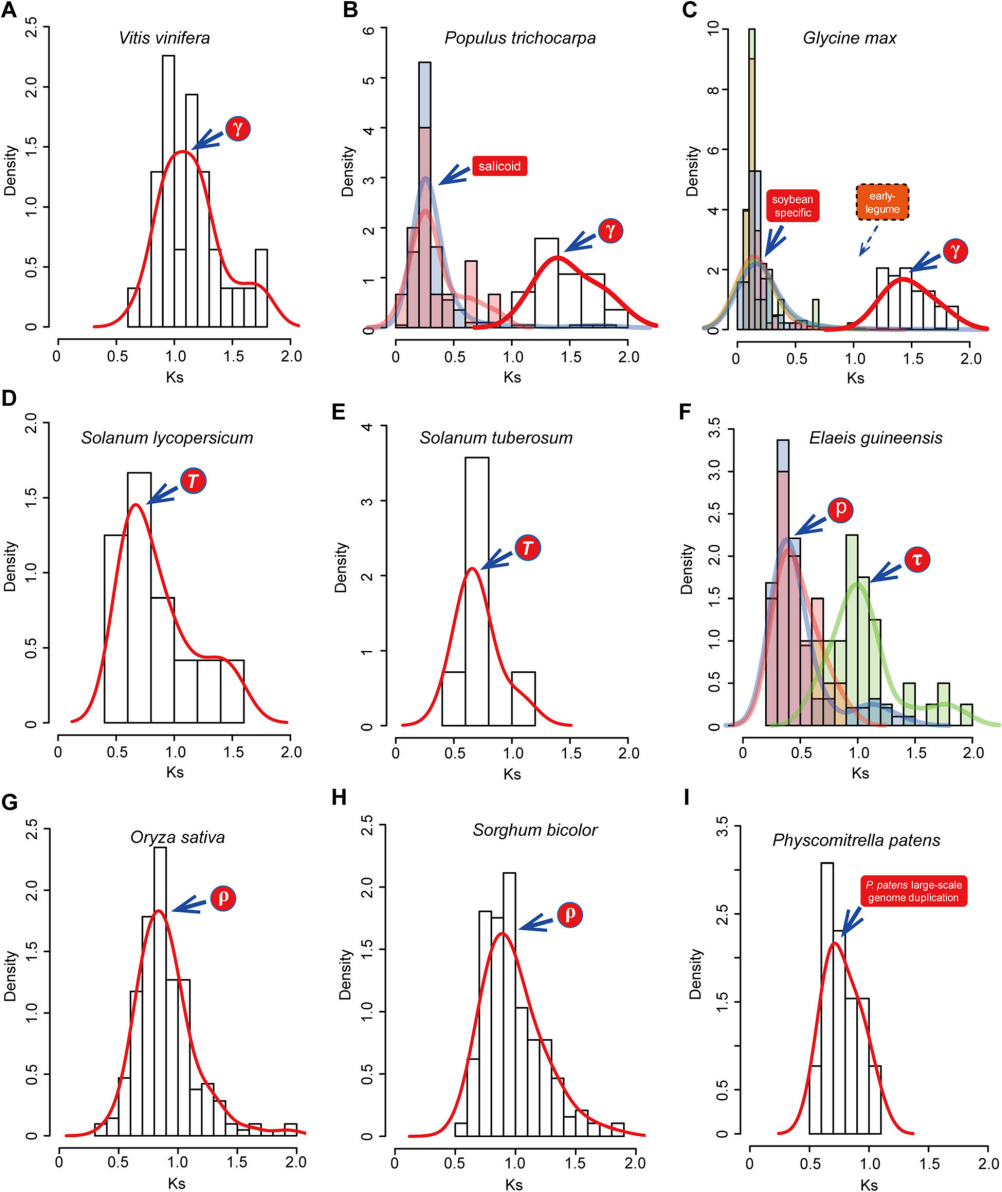
*Ks* distribution for multiple polyploidy events in different plant lineages calculated from the paralogous pairs located on the *VOZ*-containing genomic syntenic blocks. *Ks* peaks derived from the analysis of paralogous pairs on syntenic blocks surrounding the *VOZ* gene loci and the corresponding polyploidy events are indicated for individual key species: (**a**) The core-eudicot γ paleopolyploidy event was traced by analyzing paralogs in the *VOZ*-containing syntenic blocks in *Vitis* genome. (**b**) The γ paleopolyploidy and the “salicoid” events were captured using the syntenic blocks in the *Populus* genome. (**c**) The γ (red line) and “soybean-specific” (shaded light red/purple/green) duplicated syntenic blocks were conserved in the soybean genome, whereas the synteny of “early-legume” duplications (dashed box) were lost. (**d** and **e**) Identification of the *T* polyploidy event by analyzing the *VOZ*-containing syntenic blocks in the genomes of tomato and potato. (**f**) Both the precommelinid τ polyploidy (shaded green) and subsequent independent *P* duplication events (shaded light purple/red) were identified by analyzing the syntenic blocks in the genome of oil palm. (**g** and **h**) Identification for the pan-grass ρ polyploidy event by analyzing the syntenic blocks in rice and sorghum genomes. (**i**) The *VOZ*-containing syntenic blocks were identified as a component of the “large-scale genome duplication” for the *Physcomitrella patens* genome

The genomic synteny comparisons using *VOZ* gene loci as anchors together with the phylogenic tree allowed us to indicate the presence of several duplication events, but whether they precisely correspond to specific WGD events requires further supporting evidence in the form of molecular dating estimation analyses. In attempt to increase the resolving power of our analysis, adjacent duplicated genes (paralogs) that reside in sister *VOZ*-containing syntenic blocks (i.e. syntelogs, syntenic homologous genes) were employed to scrutinize *Ks* value distributions and calculate the 95% confidence interval of the mean instead of using the *Ks* values for paralogous *VOZ* genes alone. To validate the WGD events with molecular dating evidence, comparisons of peak *Ks* values were conducted to match with the corresponding events (Table 1 and Fig. 5).

**Table 1:** Comparison of peak *Ks* values for syntenic blocks flanking VOZ loci and corresponding WGD events

Species	WGD events	Ks peaks		References^d^
		VOZ-synteny block^a^	WGD Ks peak	
*Vitis vinifera*	Gamma	1.05–1.25	1.22 (0.16)^b^	[14]
*Populus trichocarpa*	Gamma	1.35–1.64	1.54 (0.24)^b^	[14]
	Salicoid	0.30–0.38	~ 0.27(0.15–0.4)	[8, 14]
*Glycine max*	Gamma	1.43–1.54	~ 1.5^c^	[17]
	Early-legume	na	0.40–0.80	[17]
	Soybean	0.19–0.22	0.06–0.39	[17]
*Solanum lycopersicum*	*T*	0.67–1.07	0.4–1.0	[8, 19]
*Solanum toberosum*	*T*	0.55–0.86	0.4–1.0	[8]
*Elaeis guineensis*	Tau	0.96–1.16	~ 1.13	[21]
	*P*	0.33–0.40	~ 0.36	[21]
*Oryza sativa*	Rho	0.85–0.90	~ 0.86 (0.6–1.0)	[8, 21]
*Sorghum bicolor*	Rho	0.94–1.01	0.6–1.3	[8]
*Physcomitrella patens*	Pp-WGD	0.69–0.87	0.5–0.9	[29]

^a^*Ks* values were presented as the range for 95% confidence interval of the mean

^b^Data depicted as median (variance) by *Tang* et al. 2008

^c^Detailed data or plot not shown in the paper, authors mentioned in the main text

^d^[8]-Vanneste et al. 2014; [14]-Tang et al. 2008; [17]-Schmutz et al. 2010; [19]-The Tomato Genome Consortium, 2012; [21]-Jiao et al. 2014; [29]-Rensing et al. 2008

To validate the γ event, the *Ks* values frequency distribution of 31 duplicated genes flanking the VOZ loci in the syntenic blocks in *Vitis* genome were investigated (Fig. 5a). Coincident with previous reports in the literature, the γ paralogs in *Vitis* genome showed a *Ks* peak of approximately 1.03 to support the core eudicot-wide duplications, a peak of 1.31 to support the eudicot-wide duplications [11], and a gamma peak around 1.2 in *Vitis* were also reported [12, 14]. For the duplicated genes in the *VOZ*-containing syntenic blocks in *Vitis*, a conspicuous *Ks* peak around 1.15 (95% CI: 1.05–1.25) was observed, suggesting this syntenic block constituted a component of the γ event (Table 1). Based on this *Ks* age estimation and considering variations in divergence rate of different paralogs, together with the genomic synteny results (Fig. 3), the core-eudicot duplication of *VOZ* transcription factor family was confirmed as product of the γ event with both spatial and temporal evidences.

The *Ks* peaks for the paralogous genes on the VOZ1- and VOZ2-anchored syntenic blocks in the poplar genome (Fig. 5b and Table 1) were averaged at 1.496 (95% CI: 1.35–1.64), a higher value than that observed for *Vitis*, perhaps suggesting an overall faster divergence rate postdating the γ event. As a polyploidy event shared with *Salix,* the “salicoid” duplication event has been reported in the poplar genome [14, 15] and it was evident that the quadruplicate *VOZ* gene loci in poplar were generated simultaneously as evidenced by the overlapping of the syntelog *Ks* peaks. The peaks around 0.34 (95% CI: 0.30–0.38) are coincident with components of the post-γ “salicoid” event [8, 14].

For the soybean genome, three recurrent genomic duplication events (γ, “early legume” and “soybean specific”) were previously identified and reported [17]. For the γ triplication in the soybean genome, the adjacent duplicated genes on the syntenic genome blocks had an average *Ks* value of 1.48 (95% CI: 1.43–1.54) (Fig. 5c and Table 1). For the most recent “soybean-specific” duplication event, three overlapping *Ks* peaks around ~ 0.21 were observed for the three pairs of adjacent duplicated genes (i.e. Glyma_VOZ1A vs -1C, −1E vs -1G and -2B vs -2D), which constituted a portion of the “soybean-specific” duplication event within the corresponding *Ks* range of 0.06–0.39 [17]. The genomic synteny of “early-legume” (*Ks* peaks at 0.4–0.8, denoted with dashed box in Fig. 5c), indicates the lost duplicated syntenic genomic blocks. Similarly, the Solanaceae-wide *T* triplication event was traced using the adjacent duplicated genes on the VOZ-containing syntenic blocks in the tomato and potato genomes (Fig. 5d and e). The *T* polyploidy event was estimated to have occurred between 53 and 91 Mya [19]. In the analysis presented here, the adjacent duplicated genes flanking the *VOZ* gene loci in the tomato genome had an average *Ks* value of 0.87 (95% CI: 0.67–1.07), which are within the *Ks* range for the *T* event (Table 1) and can be translated into an estimated divergence time of 72 ± 16.9 Mya by assuming a synonymous substitution rate of ~ 6.03e-9 site/year [60], also situating the duplication into the reported estimated time interval for the *T* polyploidy event. However, in the potato genome a smaller syntenic block with only seven adjacent duplicated genes was found flanking the *VOZ* gene loci and these generated an average *Ks* value of 0.71 (95% CI: 0.55–0.86). All of the *Ks* values obtained fell into the *Ks* range of 0.4–1.0 that constituted components of the Solanaceae *T* triplication event [8].

In the monocots, echoing the core eudicot-wide γ polyploidy event and the *T* event in Solanaceae family, two parallel polyploidy events were identified by deciphering the evolutionary history of *VOZ* genes including the precommelinid τ event and the ρ WGD leading the radiation of the Poaceae. In oil palm, the τ polyploidy event was superimposed by a subsequent duplication event termed *P* which mirrored the γ-salicoid series in poplar. Similarly, by analyzing the *Ks* distribution of syntenic duplicated genes adjacent to *VOZ* loci, conspicuous *Ks* peak constituting a component of the τ event was observed with a mean value of 1.06 (95% CI: 0.96–1.16) (Fig. 5f and Table 1). This is very close to the *Ks* mode around ~ 1.13 constituting the τ polyploidy event in oil palm as reported previously [21, 26]. And the subsequent *P* duplication event in oil palm was also circumscribed by a distinctive *Ks* distribution peak with an average value of 0.37 (95% CI: 0.33–0.40), also very close to the *Ks* mode ~ 0.36 for the oil palm genome duplication [21]. In the Poaceae, the use of duplicated syntelogs flanking the *VOZ* loci in rice and sorghum, circumscribed the polyploidy event that constituted the component of the ρ WGD event [8] with mean values of 0.88 (95% CI: 0.85–0.90) and 0.97 (95% CI: 0.94–1.01) in rice (Fig. 5g) and sorghum (Fig. 5h) respectively, both of which are close to the estimated ρ peaks reported previously (Table 1) [8, 21].

However, in the gymnosperm, we used the two *VOZ* genes from Norway spruce (*Picea abies*), which is the first conifer genome reported with an amazing 20 Gb genome size, and the syntenic genomic blocks for the *VOZ* gene loci were not detectable probably because of the massive insertion of transposable elements in the large genome [27]. The pairwise *Ks* value between the *VOZ* paralogs was 0.35, which might be the product of the “Pinaceae” WGD events with a *Ks* peak around ~ 0.25 [27, 28]. In the genome of *Physcomitrella patens*, the model moss species, two *VOZ* genes were found to locate in a syntenic region which allowed for a *Ks* distribution analyses for adjacent duplicated genes that generated a peak at ~ 0.78 (95% CI: 0.69–0.87) (Fig. 5i and Table 1). This estimation is consistent with the reported WGD event in the *P. patens* genome with a *Ks* range 0.5–0.9 [29].

### Major genome duplication events were identifiable using a support-based approach

**Fig. 6:**
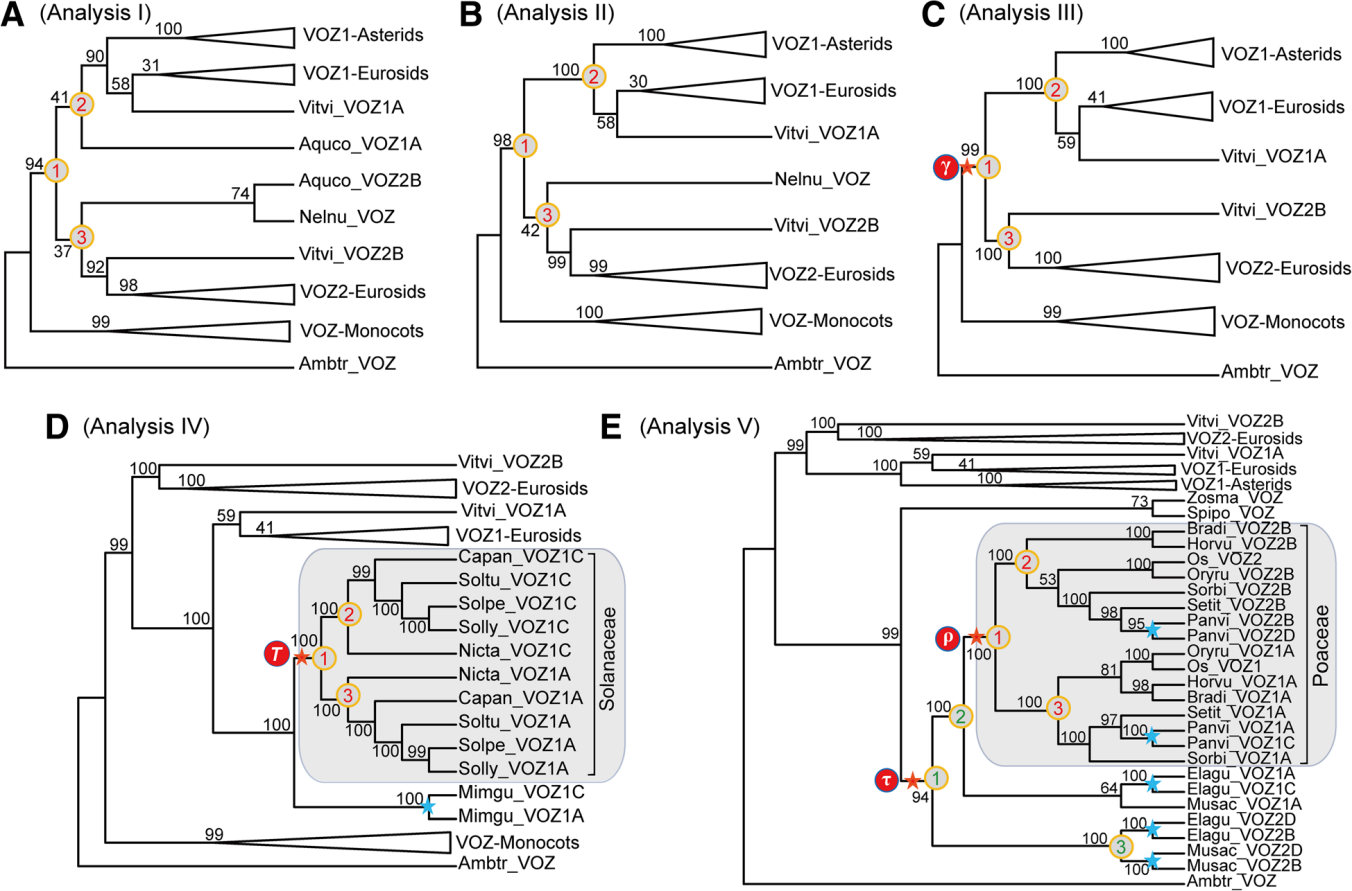
Duplication events inferred from maximum likelihood phylogenies utilizing a support-based approach. Collapsed RAxML topology and focal bootstrap values (BS) of *VOZ* transcription factor genes in different plant lineages including eudicots **a**-**c**, asterids **d** and monocots **e**, using the unique *VOZ* gene from *Amborella trichopoda* as outgroup. BS values for nodes #1, #2 and #3 indicated on the phylogenic trees were used to detect polyploidy events which are depicted in red circles. These include: the core eudicot-wide γ event (Analyses I-III); the *T* polypoildy events in Solanaceae (Analysis IV); and the precommelinid τ and pan-grass ρ duplication events (Analysis V). Red stars on the nodes of the phylogenic tree represented well-supported ancestral duplication events and blue stars denote more lineage-specific duplications

In accordance with the Angiosperm Phylogeny Group (APG) IV classification system [59], *Vitis* was used to represent the sister group to all other rosid members in the phylogenic analyses and classification of the rosid *VOZ* gene family into two clades and the two members from *Vitis* located sister to the VOZ-Rosids clade. Previously, the γ polyploidy event has been placed upon the early diversification of core eudicots and before the separation of asterids and rosids [11]. In this study, two basal eudicot species were included, sacred lotus *(Nelumbo nucifera*, Proteales) which possesses only one *VOZ* gene loci in its genome and Colorado blue columbine *(Aquilegia coerulea*, Ranunculales) which has two family members in its genome. To resolve the duplication events which could be interpreted as included in the gamma triplication, we reconstructed three independent phylogenic trees using *VOZ* genes from angiosperms with *Ambtr_VOZ* as outgroup and observed three relevant bootstrap (BS) supporting values [11] as illustrated in Fig. 6. The BS-2 and BS-3 values indicated the supporting values for VOZ1-core eudicots clade (including the *Vitvi_VOZ1A* gene) and VOZ2-rosids clade (including the *Vitvi_VOZ2B* gene), respectively and BS-1 represented the bootstrap values supporting the larger VOZ-eudicots or VOZ-core eudicots clade including both VOZ1 and VOZ2 clades.

In analysis I (Fig. 6a), genes from the two early diverging eudicots were incorporated and both BS-2 and BS-3 were lower than 50%. For analysis II (Fig. 6b), we excluded the two genes from *A. coerulea* and BS-2 (for the VOZ1-core eudicots clade) was 100%, however, BS-3 for the VOZ2-eudicots clade was below 50%. The reduced supporting value for BS-3 in analysis II was primarily a function of the location of *Nelnu_VOZ* sister to the VOZ2-rosids clade. Ultimately, in analysis III (Fig. 6c), the sequences from basal eudicots were excluded, and the duplication event occurring before the divergence of rosids and asterids was then fully supported, BS-1 was 99%, and BS-2 and BS-3 supporting the child clades were both 100%. Previous investigations proposed that Proteales and Ranunculales are outside of the γ genome triplication event [11, 12], and whole genome analyses of *Nelumbo nucifera* firmly dates the lotus-grape divergence before the pan-eudicot γ triplication [20]. However, the tree topologies generated in analyses I and II appear to support the eudicot-wide duplication of the VOZ gene family (although with some low BS support values), as also observed for a few gene families in previous studies [11, 20]. However, this may be the result of one or more of the basal eudicots contributing to a triplication event that gave rise to the core eudicot ancestor that has extant relatives (e.g. *Aquilegia* or *Nelumbo* species) that are more closely related to one of those ancestors than the ancestors are to each other. As the divergence of paralogous copies tracks the divergence of diploid species instead of the origin of the polyploid event itself, so the node for the divergence of subgenomes in a phylogeny might be older than the actual WGD event [61]. Some basal eudicot lineages might have contributed to the γ hexaploidization [20], therefore the corresponding members in basal eudicots were placed sister to the respective subgenomes in the phylogeny as depicted in analyses I and II (Fig. 6a and b).

The *VOZ* transcription factor genes in asterids were only clustered next to the VOZ1-Rosids clade and within the VOZ1-eudicots clades. As illustrated in analysis III, the *VOZ* gene duplication was fully supported as products of the γ event before the separation of asterids and rosids, but the “VOZ2-Asterids” clade does not exist at all, at least for the *VOZ* genes from lamiids (Euasterids I) which were dominantly represented by Solanaceae species presented here. This observation could be explained by intensive gene losses following the γ WGD event where only 21.6% in tomato and 14.6% in potato of the γ genes were retained from the ancestor of asterids, respectively [19]. All the asterid genomes analyzed here, like most rosids, possess two VOZ-encoding gene loci and primarily clustered as two groups designated VOZ1A-Solanaceae and VOZ1C-Solanaceae according to the nomenclature regime described above, and was depicted in analyses IV (Fig. 6d). Analysis IV confidently supported the obvious duplication event common in all Solanaceae species with BS-1, -2 and -3 values all at 100%. However, the two *VOZ* genes from *Mimulus guttatus* (currently *Erythranthe guttata,* seep monkeyflower, Phrymaceae), did not share the duplication event with the Solanaceae, as both *Mimgu_VOZ1A* and *Mimgu_VOZ1C* were placed outside of the Solanaceae clade. And similar tree topologies were reported for the *SEP1* and *SEP2* subfamilies of the MADS-Box superfamily, which assisted in revolving the independent polyploidy events between the two sister families Brassicaceae and Cleomaceae [62]. From this observation, it is highly likely that the duplication event for the VOZ1-Solanaceae clade was not a shared event for all lamiids (Euasterids I), and the two VOZ genes from *M. guttata* probably represented the products of a recently identified WGD event which was not shared with Solanaceae [63].

Because of the economic and agricultural importance of grasses, the available monocot genomes are dominated by members in the Poaceae family, however, we were able to incorporate *VOZ* genes from two commelinids, banana (*Musa acuminata,* Zingiberales) and oil palm (*Elaeis guineensis*, Arecaceae), and two Alismatales, the sea wrack (*Zostera marina*) and common duckweed (*Spirodela polyrhiza*) into the analyses. The banana genome contained three *VOZ* genes and there are four *VOZ* gene loci in the oil palm genome. The phylogenetic analysis for the monocots is depicted in Analysis-V (Fig. 6e). By focusing on the three relevant BS supporting values at critical nodes, a Poaceae-wide duplication event could be readily identified (component of the ρ WGD event), with BS-1, -2 and -3 values all at 100%. In the genome of switchgrass (*Panicum virgatum*), the analysis supports more recent species-specific duplications of *VOZ* genes that postdated the ρ duplication event and resulted in the presence of four *VOZ* gene family members in its genome. The analysis supported, from the inclusion of banana and oil palm genes, the identification of a pre-commelinid duplication event (coincident with the τ WGD event) with BS values over 90% (Analysis V, Fig. 6e). More recent lineage-specific duplications in both banana and oil palm genomes are also indicated by this analysis. The oil palm genome experienced another round of WGD (the *P* event) postdating the ancestral τ WGD event [21], and all four corresponding copies were retained and found in its genome. While three recurrent WGDs (Mγ-Mβ-Mα) were reported in the banana genome [21, 22], but only three members of VOZ genes with intact DNA binding domain were found, suggesting extensive gene losses in banana following polyploidization.

**Fig. 7:**
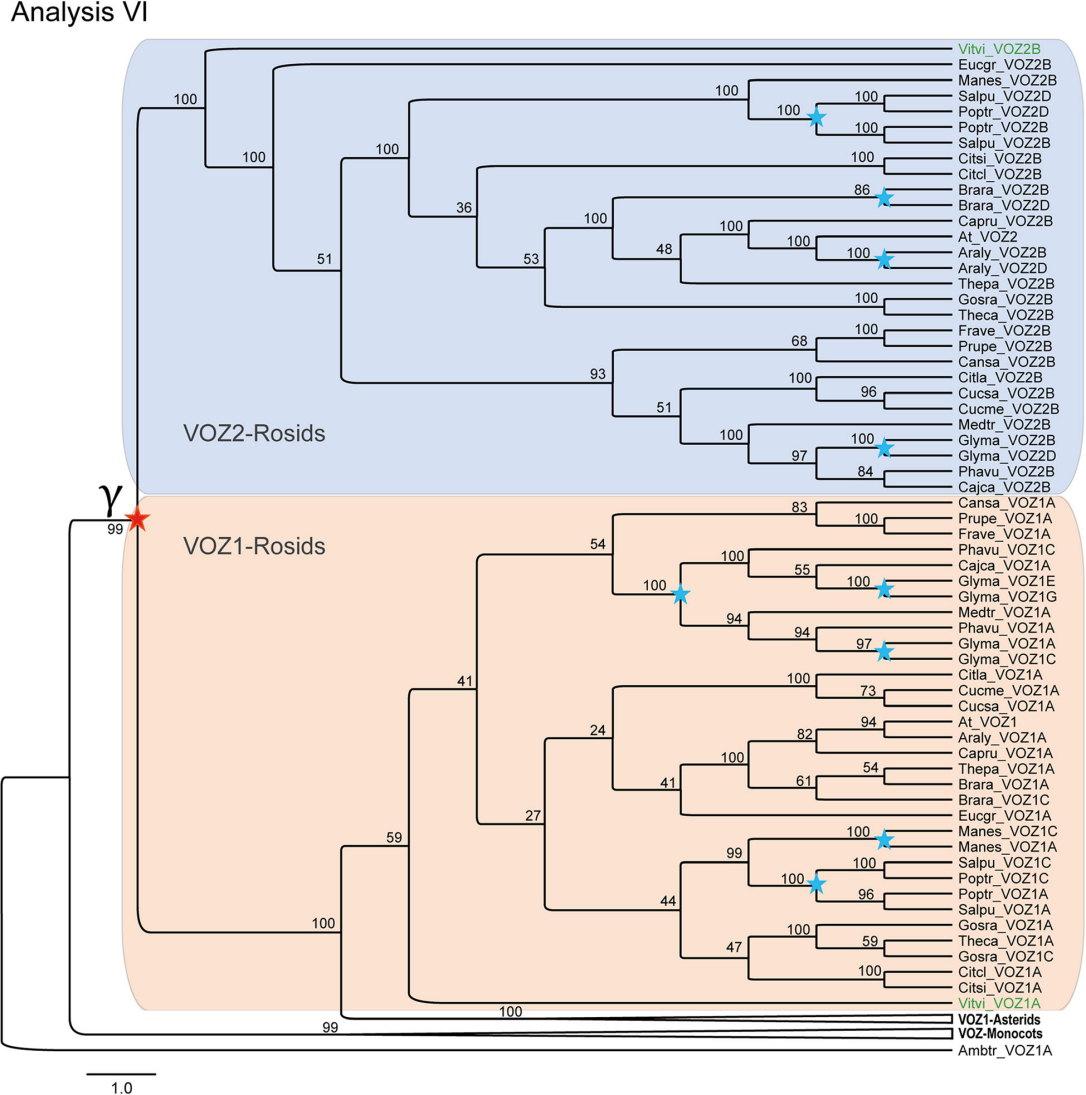
Detection of post-γ duplications within core eudicots using a support-based approach. The collapsed RAxML topology and bootstrap values (BS) of the *VOZ* transcription factor genes in flowering plants with the unique *VOZ* gene from *Amborella trichopoda* employed as outgroup. Blue stars on the nodes of the phylogenic tree represent the post-γ duplications

In most rosids, extant *VOZ* transcription factor genes constituted a dual-member gene family by retaining γ paralogs. Nevertheless, in some genomes more than two members were identified, for example poplar has four VOZ gene loci and soybean has six VOZ gene loci. We hypothesize the increase in members of the VOZ gene family to be the result of post-γ duplications in those genomes. In Analysis-VI (Fig. 7) for eurosids, using the support-based approach described above, an evident duplication event before the separation of poplar (*Populus trichocarpa*) and willow (*Salix purpurea*) was revealed. This duplication event generated two *VOZ1* and two *VOZ2* gene loci in both Salicaceae species. The duplication event may not be common for Malpighiales, because all three *VOZ* genes in cassava (*Manihot esculenta,* Euphorbiaceae), another Malpighiales species, located outside of the VOZ-Salicaceae clade [15]. In the Phaseoleae clade, the “early-legume duplication” could also be observed for the *VOZ1* subfamily and an extra round of “soybean-specific duplication” was also evident in soybean (*Glycine max*) genome, generating six VOZ gene loci (in contrast to only three loci in common bean *Phaseolus vulgaris*).

The duplication events observed in Analysis-VI, coincide with the “three paralogous peaks”, corresponding to the γ, “early-legume” and “soybean-specific” polyploidy events in the soybean genome [17]. The two *VOZ1* genes in common bean were probably generated by the post-γ Papilionoideae-wide duplication (PWGD) event, in congruence to the early-legume duplication, which was suggested to have occurred near the origin of the papilionoid lineage [16, 17]. However, in pigeon pea (*Cajanus cajan*) and barrel medic (*Medicago truncatula*), there was only one *VOZ1* gene retained.

## Discussion

The *VOZ* genes in *Arabidopsis* have previously been classified as members of a subgroup of the *NAC* transcription factor gene family [64], but sequence comparisons between *NAC* and *VOZ* genes revealed few sequence and structural similarities at the *NAC* domain and detailed inspection of the phylogenic tree including *VOZ* and *NAC* genes cannot confidently classify *VOZ* as members of the VIII-2 subfamily of *NAC* genes [64]. The functions of NAC transcription factors are primarily associated with stress responsiveness (e.g. reviewed in [65, 66]) which would also tend to set them apart from the *VOZ* genes that primarily play a role in flowering time regulation. This is highlighted by the observation that there are no *NAC* transcription factor genes found in the FlOR-ID database [49]. In both the PlantTFDB [57] and PlnTFDB [67] transcription factor databases, *NAC* and *VOZ* genes were separated into two different families. Our evolutionary data also supports the classification of *VOZ* genes as an independent transcription factor family. In concordance with the classification of the *VOZ* gene family a distinct class of transcription factors, we proposed a simplified nomenclature for individual *VOZ* genes that complies with the branch- and species-specific genomic duplication events, as described above.

**Fig. 8:**
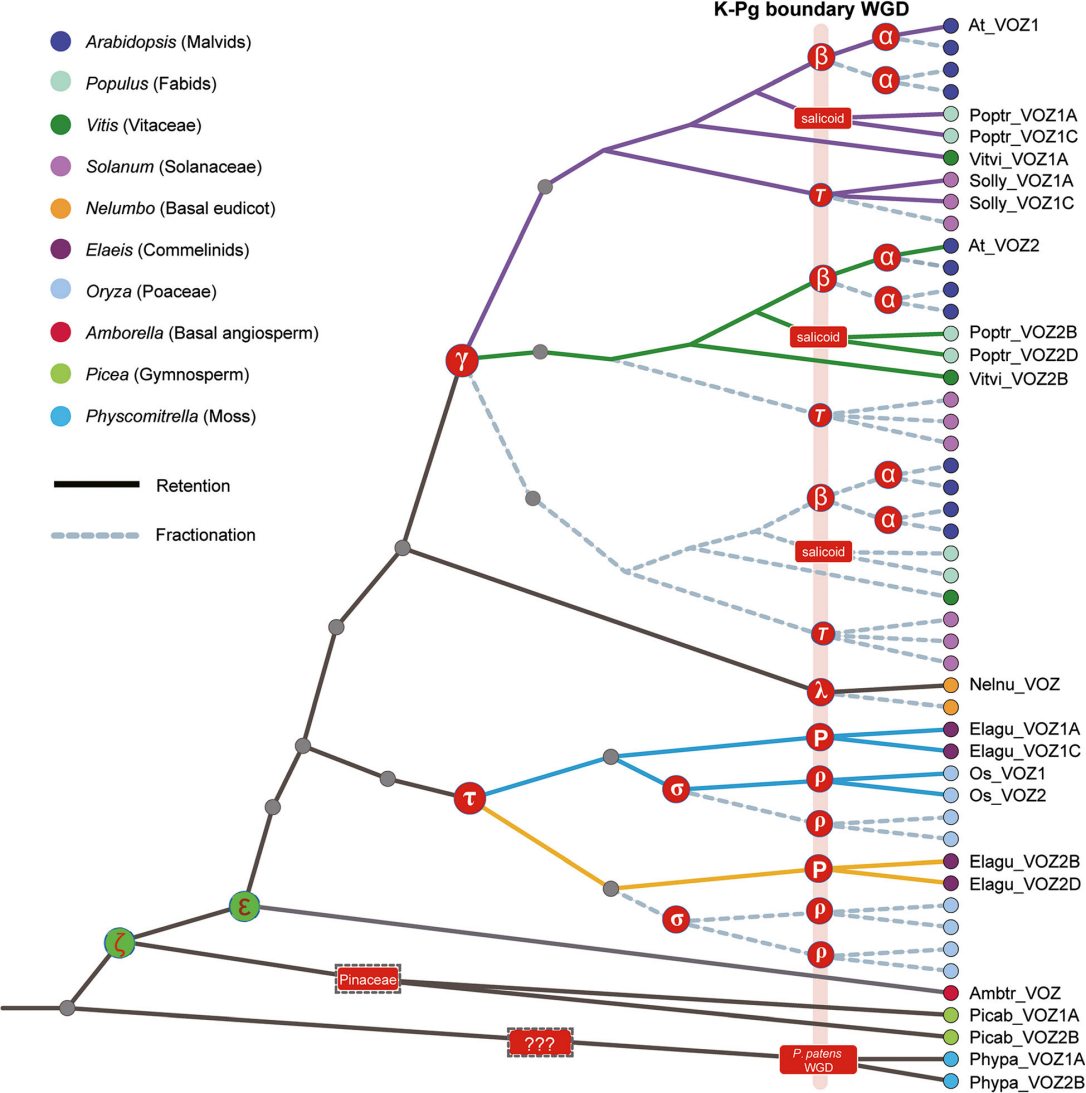
Idealized gene tree. Idealized gene family phylogenetic tree constructed to indicate gene retention and fractionation following polyploidy events in *Arabidopsis*, *Populus*, *Solanum*, *Vitis*, *Nelumbo*, *Elaeis*, *Oryza, Picea* and *Physcomitrella***.** For simplicity and illustration purposes, branch lengths are not to scale to the divergence time and the known K-Pg boundary polyploidy events are aligned and highlighted. Extant *VOZ* gene loci were labeled and the observed fractionation following polyploidization are represented as dotted lines in the gene tree. The two ancestral polyploidy events **ε** (in angiosperms) and ξ (in seed plants) are indicated as green circles

Our analyses demonstrated that not only the *VOZ* gene loci per se but the adjacent genomic synteny were highly conserved in different plant lineages throughout evolutionary history. The expansion/duplication of the *VOZ* gene family was demonstrated to be tightly associated with historical polyploidy events that occurred throughout the land plant phylogeny. Previous studies have utilized MADS-Box genes as markers for phylogenetic and molecular dating to resolve polyploidy events, particularly for shared GAMMA events on the core-eudicots [12]. Like the *VOZ* gene family, the MADS-Box gene family is also functionally associated with flowering, more so in flower development whereas *VOZ* genes have a role in the control of flowering time [8, 12, 52, 54]. The parallel and simultaneous doubling or tripling of members in both the VOZ and MADS-Box gene families, followed by biased diploidization (Fig. 8), allowed for the evaluation of the impact of ancient polyploidization for not only the morphological diversity of flowers in different plant lineages [8, 12] but also the accelerated radiation of plant species [68]. The retention of the GAMMA event derived duplicates of *VOZ* genes was highlighted in every rosid species. This polyploidy event occurred in the upper Cretaceous period and is tightly associated with the rapid radiation of eudicot species, which was addressed in Darwin’s “abominable mystery” [69]. Similarly, the gene duplications in *VOZ* family in the Solanaceae and Poaceae closely track the *T* and ρ events that subsequently triggered species radiation in these two lineages. The expansion/duplication of the *VOZ* gene family is also associated with ancestral polyploidy events in the Pinaceae as evidenced in our analysis of two members in Norway spruce, because the *VOZ* gene family duplication were very closely related in time to the Pinaceae polyploidy event, even though, in this case, we cannot find evidence in genomic collinearity assessments. The moss *Physcomitrella patens* also retained two *VOZ* genes, which we conclude to be products of the K-Pg WGD event [8] reported for this lineage, however, duplicates are not detectable for the more ancient moss-wide WGD reported in a recent study [70].

With the exception of the two most ancient ξ and ε events, whole genome analysis indicates that the *Amborella* did not experience further ploidy changes [71]. The *Amborella* genome was estimated to have evolved at a slow rate and if we estimate the rate using the 1.975 *Ks* peak that corresponds to 192 million years (5.14e-9 site/year), or the 2.764 *Ks* value that corresponds to 319 million years (4.43e-9 site/year) [10], then the rate of genome evolution is slower than that estimated in poplar (6.39e-9 site/year if we use the *Ks* of 1.496 corresponding to the GAMMA event that occurred 117 Mya) [11]. Different and homologous genes in syntenic regions in different species may evolve at drastically different rates [13]. This is evident when comparing *VOZ* genes in *Arabidopsis* to those in poplar. In *Arabidopsis*, the synonymous substitutions (*Ks*) of the two *VOZ* gene loci in *Arabidopsis* (*At_VOZ1* and *At_VOZ2*) exceeds 3.0. The genomic synteny around the *VOZ* loci was also lost after two rounds (α and β) of polyploidization-diploidization, during which the genes flanking the *VOZ* gene loci were probably fractionated and reshuffled. The current *Arabidopsis* genome is considered to be the product of three rounds of chromosome condensations, creating a relatively smaller sized genome compared to its close relatives [72, 73]. The GAMMA event peak in *Arabidopsis* is also indiscernible in the *Ks* distribution plot [35]. In poplar, after an ancestral polyploidy event that occurred around 120 million years ago, not all γ triplicated genomic collinearity for the *VOZ* genes were retained. Only the *Poptr_VOZ1C* (Potri.011G060000) locus demonstrated synteny with the two *VOZ2* genes (Potri.013G123100 and Potri.019G092800). The flanking genomic region of *Poptr_VOZ1A* (Potri.004G050900) appears to have experienced a relatively faster gene fractionation process. Nevertheless, the partially retained syntenic genome blocks provided us the chance to trace and probe these events. Similar situations could also be observed in monocots, the nucleotide evolutionary rate between paralogs formed in the pre-commelinid τ WGD is 1.7 times greater in rice than oil palm [21]. Phylogenetically related species that evolved at relatively slow rates, such as grape (one WGD), poplar (two WGDs), and soybean (three WGDs), provided the genomic evidence for the identification and dating of the aforementioned ancestral polyploidy events. In the PlantTFDB database [57], there are 1276, 2466 and 3747 TF gene loci annotated in the grape, poplar, and soybean genomes respectively. The pattern of TF gene expansion and retention makes it clear that further WGD events had doubled or tripled the number of TF-encoding genes in these genomes.

It should be noted that we estimated a relatively larger mean *Ks* value for the GAMMA paralogs in poplar (1.496) than that for grape (1.153), which is inconsistent with a recent estimation in the ranking of nucleotide evolutionary rates reported as *Populus* < *Salix* < *Vitis* < *Arabidopsis* [13]. The “salicoid” peak can be calculated to have occurred at approximately 19 Mya, assuming a mean substitution rate of 9.1e-9 site/year [74, 75], or estimated to be 26.6 Mya using the 6.39e-9 site/year estimated above, but the *Populus* and *Salix* lineages were reported to have diverged 60 to 65 Mya based on evidence from the fossil record [76]. The similar discrepancy has also been discussed earlier [75] and can be summarized that the molecular clock hypothesis of a constant substitution rate across the genus *Populus* can be rejected [77]. As a strong rate shift could have occurred when traits like woody status, large size and long generation time were established that would be associated with a strong decrease in evolutionary rate [8, 78]. Estimation of absolute divergence time using a small number of paralogous *Ks* value could lead to unexpected results [24], especially when different substitution rates were assumed [79].

## Conclusions

Based on phylogenetic tree reconstructions, we identified and classified the *VOZ* transcription factor gene family into two subfamilies in a diversity of plant species and established a nomenclature congruent with both the gene tree and the occurrence of paleopolyploidy events. Phylogenetic analyses, *Ks*-based molecular dating and genome synteny network centered on the *VOZ* gene family provided consistent and robust evidence supporting the hypothesis that *VOZ* gene family members were products of the γ and *T* events in core-eudicots, the pre-commelinid τ and grass-wide ρ events in monocots, and the “recent” WGD events in the moss *Physcomitrella patens* (Fig. 8). In addition, the retention of post-γ polyploidy events in poplar (i.e. “salicoid” event) and soybean (i.e. the “early-legume” and “soybean-specific” events) generated additional *VOZ* gene members. As a result of extensive gene losses, only two *VOZ* genes from the γ whole genome triplication event were retained in core-eudicots, and in *Arabidopsis,* copies derived from the more recent α and β WGD events were not detected. In Solanaceae and grasses, instead of retaining the more ancient γ or τ duplicates, *VOZ* gene family members were products of the more recent K-Pg boundary polyploidy events (*T* event for Solanaceae and the ρ event for grasses) (Fig. 8). Finally, we presented an idealized gene tree based on *VOZ* genes evolution and known paleopolyploidy events that demonstrate its evolutionary trajectory with clear gain-and-loss (i.e. retention-and-fractionation) patterns following WGD events in different lineages (Fig. 8), which could potentially be adopted for all other duplicated gene loci in these plant lineages. Although a small gene family, in comparison to the MADS-Box gene family in plants, the *VOZ* gene family provided concise and robust evidence for the establishment of WGD events in the land plant phylogeny. We suggest that *VOZ* duplications not analyzed in this study, but generated as more plant genomes are sequenced, will provide evidence for the existence of further polyploidy events and will complement the information gleaned from the study of the phylogeny of MADS-Box genes.

## Methods

### Data source for VOZ gene family

For precise identification of VOZ transcription factor sequences, a Hidden Markov Model (HMM) profile was built from the DNA-binding domain [51] using the VOZ protein sequences in *A. thaliana*, *V. vinifera*, *O. sativa* and *P. patens*. Sequences were retrieved from the PlantTFDB database [57] and a multiple alignment was conducted using MAFFT (v7.310) [80]. Subsequently, the alignment was manually curated to obtain the VOZ DNA-binding domain (~ 217 aa in length) and a HMM profile was created by hmmbuild in the HMMER package (version 3.1) [81]. A total of 46 taxa with available genomes were selected to represent major linages in Viridiplantae, and species phylogeny was generated based on the APG IV taxonomy [59]. Sequence data were downloaded from Phytozome (v12.1) or obtained directly from the PlantTFDB v4.0 databases [57], further compared with NCBI records if available (listed in Additional file 1: Table S1), only the longest (primary) transcripts for alternatively spliced isoforms of *VOZ* genes were retained for further analyses. To guarantee reliable sequence alignments and phylogeny reconstructions, a final inspection was conducted to eliminate protein sequences with only partial coverage of the conserved VOZ DNA-binding domain.

### Gene family phylogeny

VOZ transcription factor protein sequences were aligned using MAFFT (v7.310) [80] with the --auto option to activate the slower and more accurate L-INS-i algorithm. Corresponding coding sequences were forced onto the aligned amino acid sequences and then coding sequence alignment was trimmed using TrimAL (v1.4) [82] with the automated1 option to activate heuristic selection for reliable and conserved alignment columns which was optimized for Maximum Likelihood (ML) phylogenetic tree reconstruction. Prior to phylogenic tree construction, the alignments were subjected to a model selection procedure where various nucleotide substitution models were tested using jModelTest (v2.1.10) [83] based on the Akaike Information Criterion (AIC). Maximum likelihood phylogenetic trees were constructed using RAxML (v8.2.10) [84] under the recommended GTR + G + I substitution model (-m GTRGAMMAI) with 1000 bootstrap replicates to obtain the confidence values for interior branches of the tree. To accelerate the computational process, the Pthreads version (raxmlHPC-PTHREADS) was used. Bayesian inference phylogenic analyses were performed using MrBayes v3.2.6 [85] with two sets of four simultaneous chains (three cold and one heated, default setting in MrBayes) and ten million generations, with trees sampled every 1000 generations, under the GTR + G + I model (Lset nst = 6 rates = invgamma). The first 25% of the sampled trees were discarded as burn-in and the remaining 75% were used to generate the consensus tree and calculate the Bayesian posterior probabilities (PPs). To ensure the Bayesian MCMC runs were sufficient to reach convergence, Tracer v1.6 (http://tree.bio.ed.ac.uk/software/tracer/) was employed to analyze the trace files to ensure the Effective Sample Size (ESS) was larger than 200 and the Potential Scale Reduction Factor (PSRF) was equal to or very close to one. The phylogenic trees were reconstructed using the ML and BI methods and were visualized and edited in FigTree v1.4 (http://tree.bio.ed.ac.uk/software/figtree/).

### Synonymous substitution (*Ks*) calculations and molecular dating of Syntenic blocks

To estimate the relative divergence time of the *VOZ* genes in different lineages, the *VOZ* genes were employed as anchors to query the Plant Genome Duplication Database (PGDD) [86] with ColinearScan [87] employed with an E-value <1e-10 as the significance cutoff to obtain genomic syntenic blocks. Lists of homologous pairs were simultaneously obtained from MCScan [14] analysis. For each pair of the paralogs retained in the syntenic blocks, protein sequences were aligned using ClustalW and alignments were back translated into codon alignments using the perl script PAL2NAL [88]. Finally, the Nei-Gojobori algorithm [89], implemented in the PAML package [90], was employed to calculate paralogous *Ks* values. Paralogous pairs with *Ks* values > 2.0, suggesting saturated substitutions at synonymous sites, were excluded from subsequent analyses. *Ks* values for gene pairs with average GC contents > 75% at the third positions of codon were considered unreliable and discarded in both the rice and sorghum analyses [21, 25]. The 95% confidence interval (CI) of the mean for syntenic paralogous *Ks* values were calculated to estimate the divergence age and the corresponding polyploidy events were inferred through comparisons with previous reports (e.g. [8, 11, 21, 29]). Since the paralogous pairs on genomic syntenic blocks were presumed to be products of the corresponding WGD event, the Kernel Density Estimation (KDE) for *Ks* distributions were employed in the R statistical environment to capture the conspicuous single peaks for each polyploidy event. Based on the syntenic relationships of *VOZ* genes within and between plant genomes, the comprehensive collinearity network was constructed and illustrated in Cytoscape (v3.4) [91].

## Additional files

Additional file 1: **Table S1.** List of *VOZ* genes analyzed in each plant genome. (XLSX 18 kb)

Additional file 2: **Figure S1.** Phylogenic tree of the plant *VOZ* transcription factor genes using the Bayesian Inference method. Numbers on branches of the phylogenic tree are posterior probability support values. Branches are drawn to scale and length of the scale bar denotes 0.1 nucleotide substitutions per site. (PDF 298 kb)

Additional file 3: **Figure S2.** Patterns of coding region and intron structures of *VOZ* genes in representative plant species. The coding regions of each gene were plotted as blue boxes and introns as grey lines. (PDF 141 kb)

## Abbreviations

CI: Confidence interval
HMM: Hidden Markov Model
*Ks*: Synonymous substitutions per synonymous site
TF: Transcription factor
VOZ: Vascular plant One Zinc-finger transcription factor
WGD: Whole Genome Duplication.
