# Supplementary figures and images for: Evolution by duplication: paleopolyploidy events in plants reconstructed by deciphering the evolutionary history of VOZ transcription factors
Source: BMC Plant Biol. 2018 Oct 26;18:256. doi: 10.1186/s12870-018-1437-8 (PMC6204039; doi:10.1186/s12870-018-1437-8)

# Basal eudicots

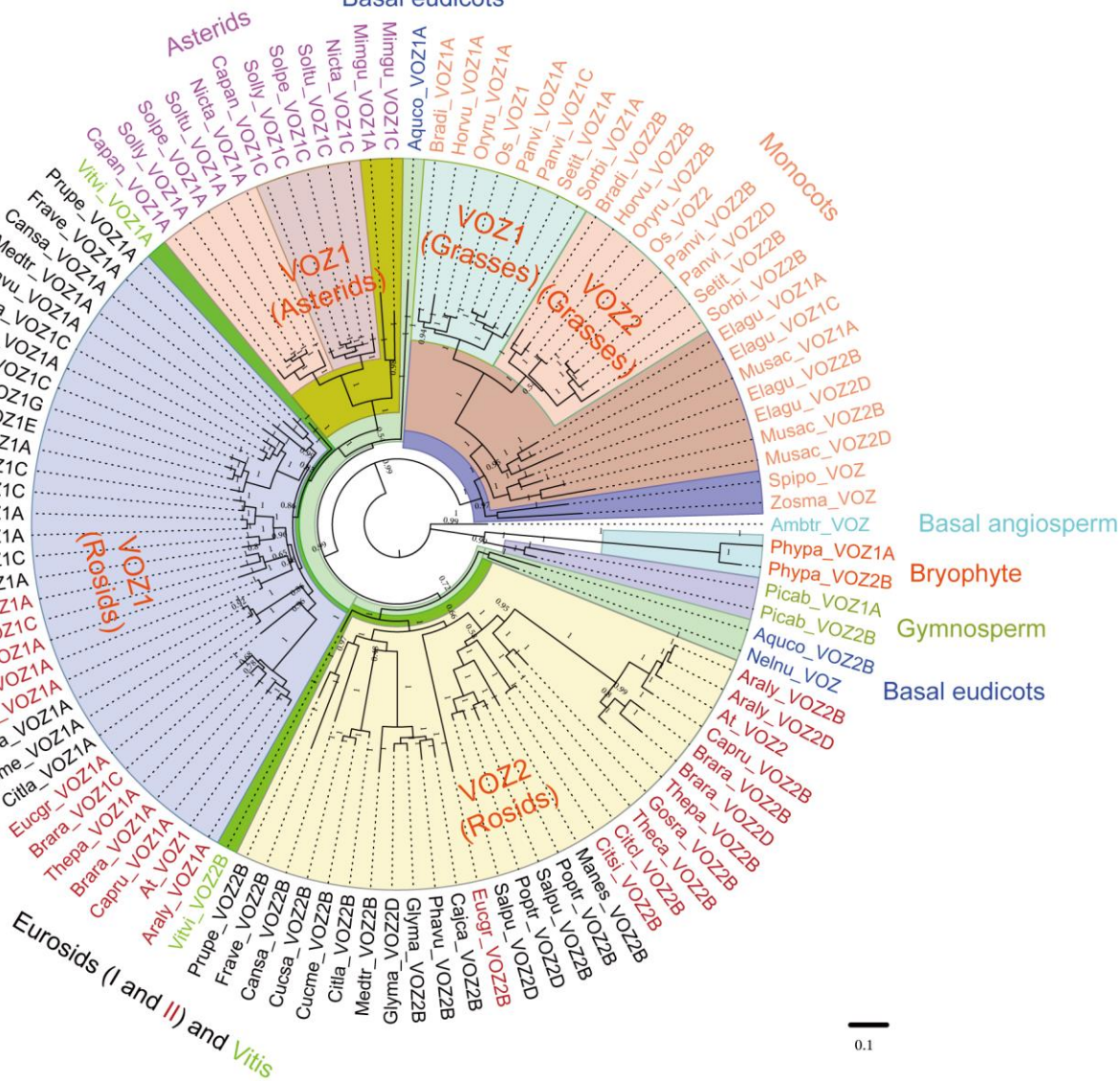

Supplement: Supplementary file 2 — Figure S1. Phylogenic tree of the plant VOZ transcription factor genes using the Bayesian Inference method. Numbers on branches of the phylogenic tree are posterior probability support values. Branches are drawn to scale and length of the scale bar denotes 0.1 nucleotide substitutions per site. (PDF 298 kb) [file 12870_2018_1437_MOESM2_ESM.pdf]

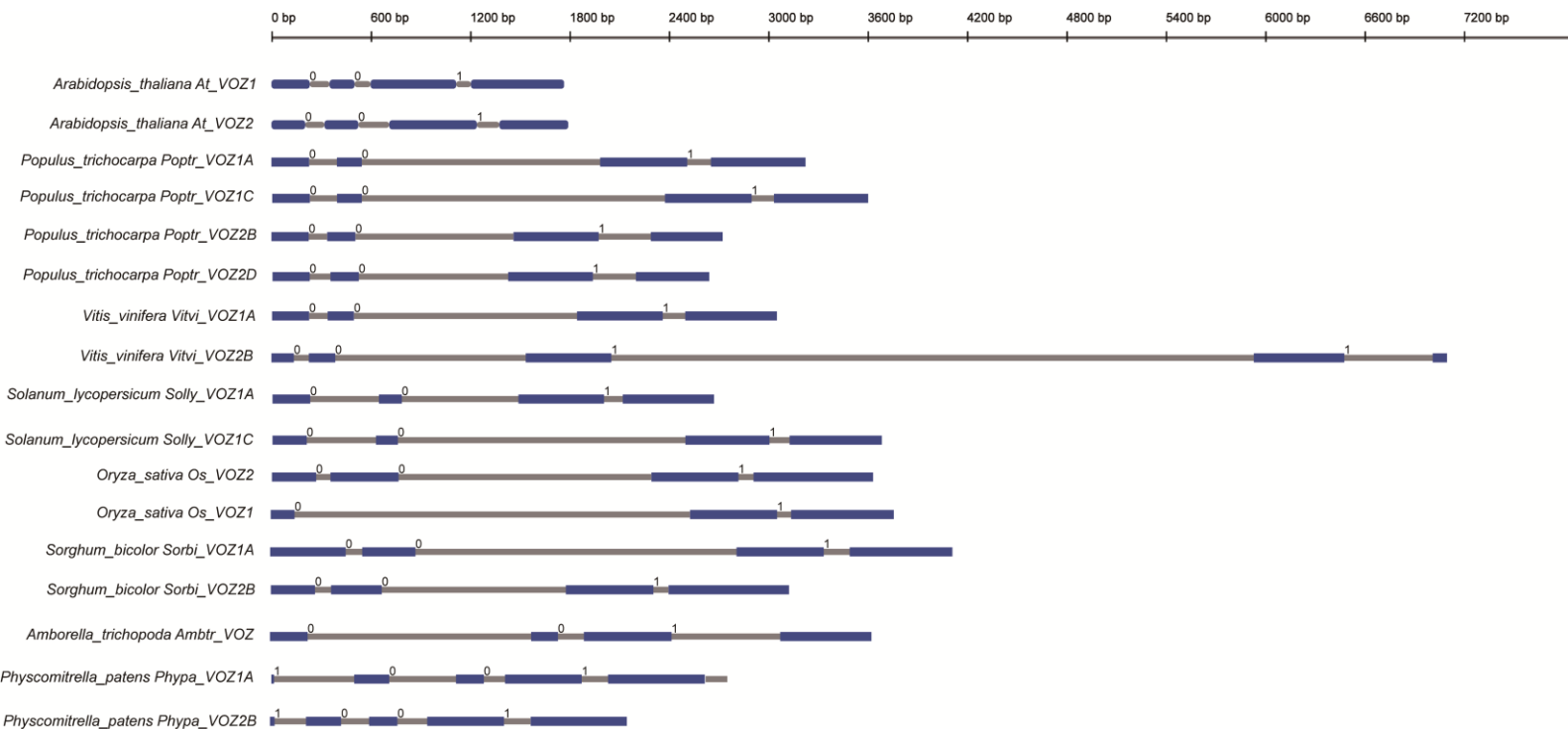

Supplement: Supplementary file 3 — Figure S2. Patterns of coding region and intron structures of VOZ genes in representative plant species. The coding regions of each gene were plotted as blue boxes and introns as grey lines. (PDF 141 kb) [file 12870_2018_1437_MOESM3_ESM.pdf]
